# Association in a Chinese population of a genetic variation in the early B-cell factor 1 gene with coronary artery disease

**DOI:** 10.1186/s12872-017-0489-2

**Published:** 2017-02-10

**Authors:** Yafei Li, Zhiyong Xie, Lei Chen, Jianjun Yan, Yao Ma, Liansheng Wang, Zhong Chen

**Affiliations:** 1 Department of Cardiology, The First Affiliated Hospital of Nanjing Medical University, No. 300 Guangzhou Road, Gu Lou Area, Nanjing, 210029 China; 2 Department of Cardiology, Xuzhou Medical University, NO.209 Tongshan Road, Xuzhou, 221000 China; 3 Department of Cardiology, Shanghai Jiao Tong University Affiliated Sixth People’s Hospital East Campus, No. 222 Huanhu Xisan Road, Pudong New Area, Shanghai, 201306 China

**Keywords:** Early B-cell factor 1, Genetic polymorphism, Coronary artery disease, Risk assessment

## Abstract

**Background:**

Early B-cell factor 1 (EBF1) is a transcription factor expressed primarily during early B cell development. Previous studies have shown EBF1 regulates blood glucose and lipid metabolism in mice with diabetes and central adiposity. Recently, a genetic variation (rs36071027) located in an EBF1 gene intron was associated with carotid artery intima-media thickness. However, whether this polymorphism is actually linked with coronary artery disease (CAD) and its severity remains unclear.

**Methods:**

This study includes 293 CAD cases and 262 controls without CAD. All participants were devided into two groups based on their coronary angiography results. A polymerase chain reaction-ligase detection reaction was used to identify genotypes at rs36071027, and CAD patients were further divided into subgroups with one-, two-, or three-vessel stenosis reflective of CAD severity.

**Results:**

The frequency of the rs36071027 TT genotype was significantly higher in CAD cases versus controls (4.8% vs. 1.5%, 95% CI: 1.13-10.81 *P* = 0.029). Subjects with a variant genotype T allele had an increased risk of CAD compared to C allele carriers (additive model: 95% CI: 1.13-2.23, *P* = 0.008). After adjustment for cardiovascular risk factors, analysis of the additive and dominant models involving rs36071027 also revealed that T allele carriers had a significantly higher risk for CAD than C allele carriers (additive model: OR 1.56, 95% CI 1.10–2.22, *P* = 0.013; dominant model: OR 1.60, 95% CI 1.07–2.41, *P* = 0.023). Furthermore, both diabetes and the CT + TT rs36071027 genotype were significantly associated with three-vessel stenosis.

**Conclusion:**

Our results in a Chinese population suggest that the TT genotype and T alleles in rs36071027 in the EBF1 gene are associated with an increased risk of CAD and its severity.

**Electronic supplementary material:**

The online version of this article (doi:10.1186/s12872-017-0489-2) contains supplementary material, which is available to authorized users.

## Background

Coronary artery disease (CAD) is one of the most common cardiovascular diseases, and myocardial infarction (MI), as its main complication, is the major cause of morbidity and mortality in China [1]. Risk factors for atherosclerotic disease include chronic inflammation, immunology, and genetic and environmental factors, all of which interact with each other to promote the formation of atherosclerosis and atherosclerotic cardiovascular diseases [2]. Over the past decade, accumulating evidence from genome-wide association studies (GWAS) has identified a series of genetic susceptibility loci associated with the risk of CAD and MI [3–6].

Early B-cell factor 1 (EBF1) located on human chromosome 5q34 is primarily expressed in early B cells, adipocytes, and olfactory neurons [7]. As a transcription factor, EBF1 was initially confirmed as a necessary factor for the maturation of B lymphocytes [8] that can activate and repress gene expression [9]. More recently, inflammation and insulin signaling were shown to be regulated by EBF1 in adipocytes, and EBF1 acts as a key integrator of signal transduction, chronic inflammation and metabolism [10]. Moreover, inconspicuous lipodystrophy, hypotriglyceridemia, and hypoglycemia have also been identified in EBF1 knockout mice [11]. Meanwhile, many EBF1 target genes have been associated with intima-media thickness (IMT) of carotid artery, a marker of subclinical atherosclerosis with high heritability [12]. Although imperfect, there are data supporting the hypothesis that EBF1 plays a critical regulatory role in metabolism and is an independent risk factor for CAD [13–16].

To our knowledge, no study has reported an association between the single nucleotide polymorphism (SNP) rs36071027 in the EBF1 gene with the risk of CAD and its severity. To increase our understanding of the functions of EBF1 in CAD and to improve our ability to predict CAD risk earlier than that provided by current clinical variables, we investigated the potential association between the rs36071027 polymorphism, which is strongly associated with carotid IMT, and the risk of CAD and its severity in a Chinese population.

## Methods

### Study subjects

The study was performed on a total of 555 unrelated individuals, which was composed of 293 CAD patients and 262 non-CAD subjects. All participants were enrolled from the First Affiliated Hospital of Nanjing Medical University between January 2013 and December 2015, and all underwent cardiac catheterization for clinical diagnosis of CAD, including angina pectoris and prior or acute MI. CAD was defined as a luminal narrowing > 50% in at least one main coronary artery. Patients with CAD were further divided into one-, two-, and three-vessel stenosis subgroups to reflect the severity of CAD, and the control subjects were identified as those with <20% luminal narrowing in any main coronary artery [17, 18]. Exclusion criteria included those with concomitant diseases, such as congenital heart disease, renal failure, and malignancies. Subjects younger than 18 years were also excluded from the study. The present study was approved by the ethics committee of the First Affiliated Hospital of Nanjing Medical University, and the consent form was signed by each participant.

### Definition of cardiovascular risk factors

Hypertension, diabetes, smoking, and dyslipidemia are well established independent risk factors for CAD. Patients with a systolic blood pressure ≥ 140 mmHg and/or a diastolic blood pressure ≥ 90 mmHg or taking hypertension-lowering medicine were diagnosed with hypertension. Diabetes was defined as having two measurements of fasting blood glucose > 7.0 mmol/L or a random glucose >11.1 mmol/L. Dyslipidemia was defined as having a total cholesterol level > 5.72 mmol/L and/or triglycerides > 1.70 mmol/L or the patient was under treatment with lipid-lowering drugs. Smoking was defined as smoking continuously or over consecutive periods at least six months.

### SNP genotyping

Blood samples were collected from each subject after overnight fasting for lipids and glucose detection, and DNA was extracted from cells using the AxyPrep DNA Blood kit (Axygen Scientific Inc, Union City, CA, USA) and stored at −80 °C until use. Genotyping for the SNP rs36071027 was conducted using the polymerase chain reaction-ligase detection method described previously [19, 20].

### Statistical analysis

Statistical analysis was conducted with the SPSS software version 17.0 (SPSS Inc., Chicago, IL, USA). Continuous variables were expressed as the mean ± standard deviation (SD) and the difference between continuous variables was calculated by the Student’s *t* test. The allele distribution and qualitative variables expressed as frequencies were compared using the chi-square (*χ*2) test. For both CAD and control groups, a *χ*2 goodness-of-fit test was performed with the Hardy–Weinberg equilibrium. An odds ratio (OR) and a 95% confidence interval (CI) were used to determine the correlation between the C/T polymorphism and the risk of CAD. A multiple logistic regression analysis, adjusted for factors, including age, gender, BMI, smoking, hypertension, diabetes, and dyslipidemia, was used to evaluate whether the EBF1 genetic variants and other related risk factors were independent factors for the severity of CAD. Additionally, we also used additive and dominant models to assess the association of the C/T polymorphism between the CAD and control groups. For all tests, a *P* value < 0.05 (2-tailed) was considered significant.

## Results

### Demographic information

The baseline data of the CAD patients and controls are listed in Table 1. Compared with the controls, a significantly higher proportion of patients with CAD were affected by diabetes and smoking (all *P* < 0.01). However, no significant differences were observed regarding gender composition, mean age, body mass index (BMI), or the proportion of patients affected by hypertension or hyperlipidemia between the CAD cases and controls (all *P* > 0.05). In regards to the coronary angiographic findings, 93 (31.7%), 99 (33.8%), and 101 (34.5%) CAD cases had one-, two-, and three-vessel disease, respectively.

**Table 1:** Baseline characteristics of CAD cases and controls

Variables	CAD cases (*n* = 293)	Controls (*n* = 262)	*P*
	*N* (%)	*N* (%)	
Age (years)	66.72 ± 11.10	65.14 ± 12.16	0.110
Sex (male)	163 (55.6)	125 (47.7)	0.062
BMI (kg/m^2^)	24.93 ± 3.08	25.19 ± 3.47	0.363
Smoking, n (%)	107 (36.5)	64 (24.4)	0.002
Hypertension, n (%)	100 (34.1)	79 (30.2)	0.317
Hyperlipidemia,n (%)	106 (36.2)	85 (32.4)	0.355
Diabetes, n (%)	92 (31.4)	54 (20.6)	0.004
Number of involved vessels,n (%)			
one	93 (31.7)		
two	99 (33.8)		
three	101 (34.5)		

Age and BMI are expressed as the mean ± SD and were compared using the Student’s *t*-test. Other data are expressed as frequencies and percentagesand were compared using the *χ*2-test

Abbreviations: *CAD*, coronary artery disease; *BMI*, body mass index

### Genotype frequencies and their associations with CAD

The genotype distribution of rs36071027 in the CAD cases and control subjects is provided in Table 2. The genotype distribution of rs36071027 in the cases and controls in this study showed no deviation from the Hardy–Weinberg equilibrium (*P* > 0.05). The frequency of the TT genotype of rs36071027 was significantly higher in CAD patients than that in controls (4.8% vs. 1.5%, *P* = 0.029). Compared with C genotype allele carriers of rs36071027, the T genotype allele carriers had an increased risk of CAD under the additive model (OR 1.59, 95% CI 1.13–2.23, *P* = 0.008). After adjustment for cardiovascular risk factors, analysis of the additive and dominant models involving rs36071027 also revealed that T allele carriers had a significantly higher risk for CAD than C allele carriers (additive model: OR 1.56, 95% CI 1.10–2.22, *P* = 0.013; dominant model: OR 1.60, 95% CI 1.07–2.41, *P* = 0.023). The multiple logistic regression analysis showed that individuals with a TT genotype had 2.98-fold higher risk of CAD than CC carriers (95%CI 0.94-9.46, *P* = 0.064).

**Table 2:** Association analyses of rs36071027 genotypes in the EBF1 gene between CAD patients and controls

Genotypes	CAD cases (*n* = 293)	Controls (*n* = 262)	OR (95% CI)	*P*	Multiple adjusted OR^a^ (95% CI)	*P*
	*n* (%)	*n* (%)				
rs36071027						
CC	211 (72.0)	211 (80.5)	1		1	
CT	68 (23.2)	47 (17.9)	1.45 (0.953–2.20)	0.083	1.48 (0.96–2.27)	0.074
TT	14 (4.8)	4 (1.5)	3.50 (1.13–10.81)	0.029	2.98 (0.94–9.46)	0.064
Additive model			1.59 (1.13–2.23)	0.008	1.56 (1.10–2.22)	0.013
Dominant model			1.61 (1.08–2.39)	0.019	1.60 (1.07–2.41)	0.023

Abbreviations: *OR*, odds ratio; *CI*, confidence interval

^a^Logistic regression model, adjusted by age, sex, hypertension, diabetes, smoking, body mass index and hyperlipidemia

Additive model: TT vs. CC. Dominant model: (TT + CT) vs. CC

### Gene-smoking and gene-diabetes interactions on the risk of CAD

Further subgroup analyses were performed to determine the effect of a gene-smoking and gene-diabetes interactions. As shown in Table 3, subjects with CT + TT genotypes or CC + smoking both had an increased risk of CAD (OR1.72, 95% CI 1.07-2.77, *P* = 0.025; OR1.88, 95% CI 1.23-2.86, *P* = 0.017; respectively). The smokers with the CT + TT genotype had a 1.61-fold higher risk of CAD than non-smoking subjects with the CC genotype (95%CI 1.30-5.24, *P* = 0.009). Furthermore, subjects with CT + TT genotypes + diabetes had higher risk of CAD (OR3.93, 95% CI 1.74-8.89, *P* = 0.001) than non-diabetes subjects with the CC genotype; however, the risk of CAD did not increase among non-diabetes subjects carrying CT + TT genotype (OR1.41, 95% CI 0.89-2.23, *P* = 0.142).

**Table 3:** Gene-smoking and gene-diabetes interaction in patients with CAD cases and controls

Variables		CAD cases	Controls	OR (95% CI)	*P*	adjusted OR ^a^ (95% CI)	*P*
rs36071027	Smoking						
CC	No	132 (45.1)	160 (61.1)	1		1	
CC	Yes	79 (27.0)	51 (19.5)	1.88 (1.23–2.86)	0.003	1.89 (1.15–3.12)	0.012
CT + TT	No	54 (18.4)	38 (14.5)	1.72 (1.07–2.77)	0.025	1.80 (1.11–2.93)	0.017
CT + TT	Yes	28 (9.6)	13 (5.0)	2.61 (1.30–5.24)	0.007	2.55 (1.22–5.30)	0.013
*P*_interation_					0.882		0.747
rs36071027	Diabetes						
CC	No	147 (50.2)	165 (63.0)	1		1	
CC	Yes	64 (21.8)	46 (17.6)	1.56 (1.01–2.42)	0.047	1.58 (1.00–2.49)	0.050
CT + TT	No	54 (18.4)	43 (16.4)	1.41 (0.89–2.23)	0.142	1.40 (0.88–2.23)	0.160
CT + TT	Yes	28 (9.6)	8 (3.1)	3.93 (1.74–8.89)	0.001	3.84 (1.68–8.78)	0.001
*P*_interation_					0.249		0.280

Abbreviations: *OR*, odds ratio; *CI*, confidence interval

^a^Logistic regression model, adjusted by age, sex, hypertension, diabetes, smoking, body mass index and hyperlipidemia

### The relationship of clinical characteristics and risk factors to the severity of CAD

The basic clinical characteristics and genotype frequency of rs36071027 among all subjects are presented in Table 4. Except for the distribution of sex, diabetes, and smoking, no significant differences exist with regards to age, BMI, hypertension, or hyperlipidemia among the four groups, including controls and CAD patients with one-, two-, and three-vessel stenosis. In Table 5, by using multiple logistic regression models, we analyzed the risk factors of coronary lesion severity between the control subjects and the three groups of patients with CAD. The current study revealed that only smoking, diabetes, and a CT/TT rs36071027 genotype were significant risk factors for three-vessel CAD (OR 2.04, 95% CI 1.08-3.87, *P* = 0.028; OR 2.95, 95% CI 1.76-4.95, *P* < 0.001; OR 1.96, 95% CI 1.14-3.37, *P* = 0.015).

**Table 4:** Clinical characteristics in controls and CAD patients with different vessel lesions

Variables	Number of involved vessels				*P*
	0 (*n* = 262)	1 (*n* = 93)	2 (*n* = 99)	3 (*n* = 101)	
Age (years)	66.14 ± 12.16	66.84 ± 11.93	66.36 ± 10.83	69.97 ± 10.66	0.439
Sex (male)	125 (47.7)	46 (49.5)	64 (64.6)	53 (52.5)	0.036
BMI (kg/m^2^)	25.19 ± 3.47	24.58 ± 3.04	25.37 ± 3.07	24.83 ± 3.10	0.294
Smoking,n (%)	64 (24.4)	26 (28.0)	43 (43.4)	38 (37.6)	0.002
Hypertension, n (%)	183 (69.8)	57 (61.3)	67 (67.7)	69 (68.3)	0.509
Hyperlipidemia, n (%)	85 (32.4)	32 (34.4)	33 (33.3)	41 (40.6)	0.530
Diabetes,n (%)	54 (20.6)	19 (20.4)	29 (29.3)	44 (43.6)	<0.001
rs36071027					
CC	211 (80.5)	67 (72.0)	76 (76.8)	68 (67.3)	0.068
CT	47 (17.9)	23 (24.7)	19 (19.2)	26 (25.7)	
TT	4 (1.5)	3 (3.2)	4 (4.0)	7 (6.9)	

Abbreviations: *OR*, odds ratio; *CI*, confidence interval

Age and BMI are expressed as the mean ± SD and were compared using the Student’s *t*-test. Other data are expressed as frequencies and percentages and were compared using the *χ*2-test

**Table 5:** The rs36071027 polymorphism and other risk factors in 1-,2-, and 3-vessel disease patients compared with non-CAD subjects by a logistic regression

Variables	One-vessel disease		Two-vessel disease		Three- vessel disease	
	OR (95% CI)	*P*	OR (95% CI)	*P*	OR (95% CI)	*P*
Age	1.02 (1.00–1.04)	0.117	1.01 (0.99–1.03)	0.254	1.01 (0.99–1.03)	0.347
Sex	1.00 (0.55–1.81)	0.996	0.72 (0.39–1.31)	0.280	1.10 (0.60–2.02)	0.757
BMI	0.95 (0.88–1.02)	0.170	1.02 (0.95–1.09)	0.643	0.97 (0.90–1.05)	0.469
Smoking	1.23 (0.63–2.38)	0.548	2.04 (1.11–3.76)	0.023	2.04 (1.08–3.87)	0.028
Hypertension	0.61 (0.36–1.04)	0.067	0.89 (0.52–1.50)	0.658	0.82 (0.48–1.41)	0.478
Hyperlipidemia	1.16 (0.69–1.94)	0.580	1.12 (0.67–1.86)	0.675	1.48 (0.90–2.46)	0.124
Diabetes	1.02 (0.56–1.88)	0.940	1.60 (0.92–2.78)	0.097	2.95 (1.76–4.95)	<0.001
rs36071027 (CT + TT vs. CC)	1.67 (0.96–2.91)	0.071	1.27 (0.71–2.26)	0.420	1.96 (1.14–3.37)	0.015

Abbreviations: *OR*, odds ratio; *CI*, confidence interval

## Discussion

In this study, we performed a hospital-based case–control study to investigate the potential association between the rs36071027 polymorphism and the risk of CAD and its severity. Our study demonstrated that the rs36071027 variants in the EBF1 gene in a Chinese population were significantly associated with an increased risk of CAD and its severity, which provides novel data to this field in the current era of “precision medicine” and helps improve our capacity for early CAD risk prediction.

Besides being a vital gene for the development and differentiation of B lymphocytes, EBF1 is also involved in the differentiation of adipose lineage cells [21, 22]. Studies in knockout mice have revealed a function for EBF1 in metabolism due to mouse phenotypes including lipodystrophy, hypotriglyceridemia, and hypoglycemia [11]. Compared with the wild type controls, the symptom of lipodystrophy in the EBF1 knockout mice is characterized by additional brown adipose tissue and a striking reduction in white adipose tissue in the bone marrow [11, 23]. Recently, scholars have determined that EBF1’s function in early B-cell development could be inhibited by active NOTCH signaling [24]. Moreover, the NOTCH1 signal pathway plays a critical regulatory role in the formation of unstable atherosclerotic plaques [25] and is activated in a rat model of post-acute MI [26]. These data support the role of EBF1 gene variants and the NOTCH signaling pathway in regulating metabolism of fatty acids and lipids and the formation of vulnerable atherosclerotic plaques.

In terms of previous GWAS results identifying gene variants significantly associated with cardiovascular diseases in a European population [27, 28]. The EBF1 gene has also been identified as potential critical regulatory gene for the formation of atherosclerosis and CAD [13–16]. Scholars [12] revealed that the rs36071027 variant in the EBF1 gene increases the risk of IMT, which is not only significantly associated with the severity of CAD, but is also a screening index of CAD. In the present study, the rs36071027 TT genotype frequency in CAD patients was significantly higher than in controls. Participants with the rs36071027 TT genotype or a T allele were more susceptible to CAD than those with the CC genotype or a C allele. These findings are consistent with the role of EBF1 gene variants found in previous studies.

In the present study, there is higher proportion of smoking and diabetes in the CAD group than in the control group. Smoking is well-known to be one of the main risk factors for CAD. Smoking contributes to the inflammatory process through promoting the release of inflammatory cytokines, such as C-reactive protein, interleukin-1 and tumor necrosis factor-α. Furthermore, inflammation can interact with lipoprotein metabolism and influence endothelial function [29]. In the current study, using an interaction model theory, the interaction between smoking and the genotype was used to explore gene loci and the interaction of smoking and their relationship with the risk of CAD. Interestingly, our results showed that being a carrier of the rs36071027 CT/TT genotypes or smoking both could increase the risk for CAD, although the interaction effect between the CT/TT rs36071207 genotypes and smoking on the risk of CAD was similar to either factor alone. Furthermore, only subjects with CT + TT genotypes and diabetes had increased risk of CAD compared to non-diabetes subjects with the CC genotype.

As another important risk factor for CAD, diabetes has a common environmental and genetic basis with CAD, which is the key concept of the theory of “common ground’ of CAD and diabetes [30]. Furthermore, Cutlip DE et al. found that comorbid diabetes is the strongest predictor of clinical vascular restenosis after a coronary intervention [31]. In the present study, a multivariate logistic regression model showed that diabetes and the rs36071027 CC + TT genotype have a significant association with the severity of CAD.

### Limitations

This study has several limitations. First, this was an observational study, and the sample size was relatively small, which might under-power the results of our study. Second, because of the case–control design, selection bias might affect our findings. Furthermore, since all control subjects have suspicion of having significant CAD although severe coronary stenosis was ruled out by coronary angiography, the control group does not represent a general healthy population, and more concomitant risk factors could be assumed in the control group compared to the general population. Furthermore, the present study lacks direct cause-and-effect evidence indicating whether the variations of rs36071027 in the EBF1 gene are functional or not, and the pathogenic mechanism for these variants to induce CAD has not been determined. Finally, our study sample came from a Chinese population, and applying these results to other ethnic groups should be done with caution. However, our study obviously provides valuable information to future studies connecting the EBF1 gene and CAD.

## Conclusion

This study observes for the first time that the TT genotype at rs36071027 increases the risk of CAD, and the rs36071027 CT + TT genotype can potentially be used as a gene marker to predict the severity of CAD. Our study results provide new biological insights into EBF1 variants and CAD risk and may identify novel targets for the prevention of cardiovascular disease. Nevertheless, further studies are necessary in order to fully elucidate the role of EBF1 in the pathogenesis of CAD.

## Additional file

Additional file 1: Clinical data of CAD cases and controls from 2013 to 2015. For the convenience of data analysis, Arabic numbers were used to describe the patient’s specific characteristics and the details were placed in the first line of the Additional file 1. (XLSX 40 kb)

## Abbreviations

EBF1: Early B-cell factor 1
CAD: Coronary artery disease
MI: Myocardial infarction
GWAS: Genome-wide association studies
IMT: Intima-media thickness
SNP: Single nucleotide polymorphism.
